# Radiation- and Photo-induced Activation of 5-Fluorouracil Prodrugs as a Strategy for the Selective Treatment of Solid Tumors

**DOI:** 10.3390/molecules13102370

**Published:** 2008-10-01

**Authors:** Takeo Ito, Kazuhito Tanabe, Hisatsugu Yamada, Hiroshi Hatta, Sei-ichi Nishimoto

**Affiliations:** Department of Energy and Hydrocarbon Chemistry, Graduate School of Engineering, Kyoto University, Kyoto 615-8510, Japan

**Keywords:** 5-Fluorouracil, Prodrugs, Hypoxia, Hydrated Electrons, Radiolysis, Photolysis

## Abstract

5-Fluorouracil (5-FU) is used widely as an anticancer drug to treat solid cancers, such as colon, breast, rectal, and pancreatic cancers, although its clinical application is limited because 5-FU has gastrointestinal and hematological toxicity. Many groups are searching for prodrugs with functions that are tumor selective in their delivery and can be activated to improve the clinical utility of 5-FU as an important cancer chemotherapeutic agent. UV and ionizing radiation can cause chemical reactions in a localized area of the body, and these have been applied in the development of site-specific drug activation and sensitization. In this review, we describe recent progress in the development of novel 5-FU prodrugs that are activated site specifically by UV light and ionizing radiation in the tumor microenvironment. We also discuss the chemical mechanisms underlying this activation.

## Introduction

5-Fluorouracil (5-FU) is the first class of compounds that has been subjected to intensive research as a chemotherapeutic agent. Although 5-FU is a potent radiosensitizer in colon and rectal cancers, and acts in a similar manner as 5-bromouracil, its main biochemical action is as an antimetabolite of the uracil anabolic pathways [1,2]. The cytotoxic effect of 5-FU in most systems is attributed primarily to its anabolism to 5-fluoro-2’-deoxyuridine monophosphate (FdUMP), a potent inhibitor of thymidylate synthase [3,4], a pivotal enzyme in pyrimidine biosynthesis [5,6,7]. Thymidylate synthase is a vital enzyme for the growth of tumors, and the expression of this gene is dependent on the cell cycle. 5-FU shows greater selectivity against solid tumors with multiresistance to drugs than do other antitumor agents. However, 5-FU is particularly toxic to dividing tissues, and its clinical use is limited by its severe side effects on normal cells.

The term “prodrug”, first introduced by Albert, refers to a chemically modified form of a drug [8] that is devoid of pharmacological activity, but that can be converted to the active form of the drug in a biological system, where it exerts the desired action. This strategy can improve the limitations associated with the effective transport into tumor cells, catabolic inactivation before the cytotoxic entity can reach the tumor, and short plasma half-1ife [9,10,11,12]. The prodrug strategy for a site-specific or tumor-targeting delivery has been employed, and much effort has been expended in searching for prodrugs that might improve the clinical utility of 5-FU as an important cancer chemotherapeutic agent. Examples include (1) prodrug forms of 5-FU such as tegafur (Ftorafur) [1-(2-tetrahydro-furanyl)-5-fluorouracil] derivatives [13,14,15], 1-alkylcarbamoyl-5-fluorouracils [16,17], 5-fluoro-2’-deoxyuridine (5-FdUrd) derivatives [18,19], and polymeric matrix systems for the controlled release of 5-FU [20,21,22]; (2) the recently advanced tumor-specific targeting of 5-FU prodrugs using tumor-specific gene expression such as antibody-directed enzyme prodrug therapy [23,24,25] and targeting carcinoembryonic antigen-promoted activity [26,27]; and (3) intratumoral prodrug activation in which a nontoxic drug is converted into 5-FU by intratumorally expressed enzymes [28,29].

**Figure 1:**
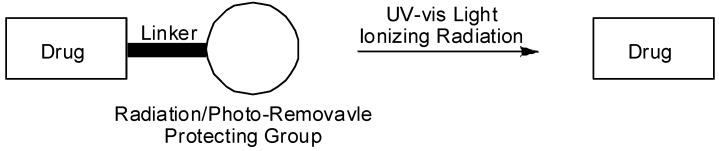
Activation of prodrugs by external triggers.

Most antitumor prodrugs including 5-FU have been designed to release the parent drug by either chemical hydrolysis or enzymatic hydrolysis. However, both processes have their drawbacks: chemical hydrolysis is not restricted to tumor tissue, and enzymatic release requires the identification of suitable tumor-specific enzymes or the targeted delivery of enzymes to the tumor tissue. To provide an alternative prodrug design for overcoming these problems, attention has focused on the external and nonenzymatic activation of prodrugs to achieve more direct control of drug release in a targeted area of the body. Among the various external triggers, ionizing radiation and UV-visible (UV-vis) light are particularly attractive because they can be controlled precisely in terms of the energy and irradiation site (Figure 1). The isolation and characterization of N1-C5’-linked pyrimidine dimer hydrates with a 5-FU component has been reported [30,31]. 5-FU dimer hydrates have also been identified as a new class of radiation-activated prodrugs that are not activated by enzymatic or spontaneous reaction processes and show no antitumor activity, but these prodrugs show selective toxicity against hypoxic tumor cells after the release of the antitumor agent of 5-FU by exposure to ionizing radiation under hypoxic  conditions. Photoinduced activation of prodrugs is achieved by photocleavage of the protecting groups in the prodrugs via direct absorption of UV-vis light. Although short-wavelength light does not penetrate through the human body, endoscopic delivery of light of a specific wavelength can activate this type of prodrug. In the current review, we summarize recent efforts to develop novel 5-FU prodrugs that are activatable by external physical agents such as ionizing radiation and UV–vis light under tumor-specific conditions.

## Radiation-activatable 5-FU Prodrugs

It is well known that various electrophilic compounds can enhance the sensitivity of hypoxic cells toward ionizing radiation [32,33]. Because of the inefficient supply of oxygen to the core of tumor tissue by poorly developed blood vessels, hypoxic cells in the solid tumors are resistant to radiation therapy and may be likewise refractory to certain types of chemotherapeutic agents operative through a variety of mechanisms [34].

Wide varieties of bioreduction-activatable prodrugs have been developed as anticancer agents [35,36,37]. Bioreductive prodrugs can be activated into their radical intermediates, which are oxidized to the prodrug under aerobic conditions. However, under hypoxic conditions (e.g., in solid tumors), this oxidation is much slower and usually increases the levels of toxic radical intermediates, resulting in a solid tumor-selective therapy. In general, hypoxic cells in low oxygen tension regions are more resistant to treatment with radiotherapy and require a two- to three-fold higher radiation dose, indicating the importance of bioreductive drugs.

**Scheme 1:**
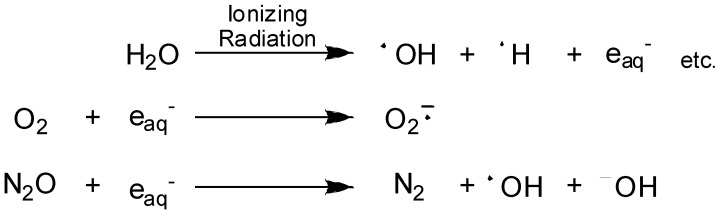
Primary water radicals generated from water radiolysis.

Radiation generates high concentrations of molecular free radicals, hydrated electrons (e_aq_^–^), hydrogen atoms (^•^H) and hydroxyl radicals (^•^OH) in irradiated tissues [38]. Under aerobic conditions, hydrated electrons react with O_2_ to produce superoxide radical anion (O_2_^–•^) (Scheme 1). In contrast, hydrated electrons can initiate reductive activation of the prodrugs under hypoxic conditions.

**Figure 2:**
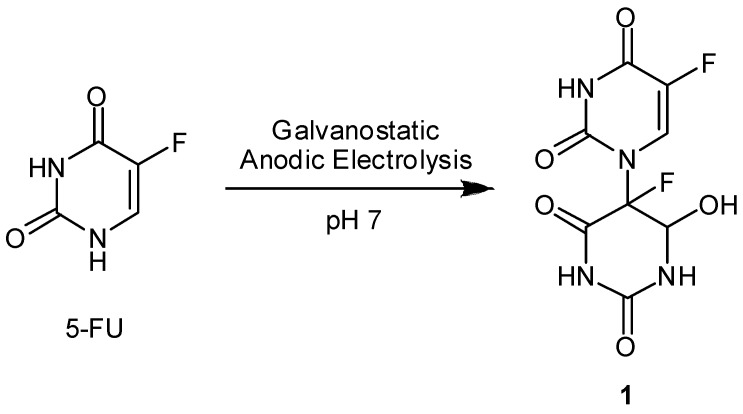
Electrochemical synthesis of N1-C5’-linked 5-FU dimer hydrate **1**.

**Figure 3:**
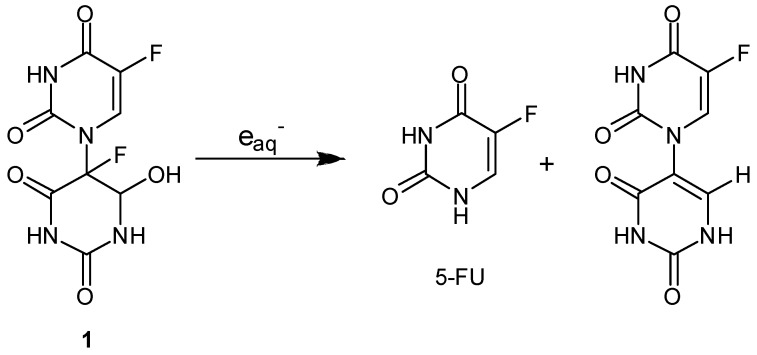
Reductive splitting of N1-C5’-linked 5-FU dimer hydrate **1** and release of 5-FU.

**Figure 4:**
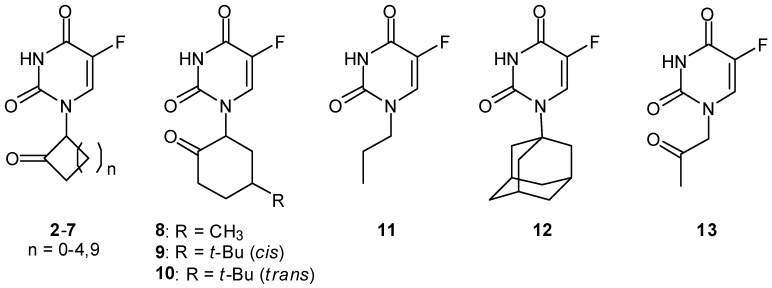
Structures of N1-substituted 5-FU prodrugs **2**-**13**.

We have reported that galvanostatic electrolysis or radiation-induced oxidation of aqueous 5-FU solution yields the N1–C5’-linked dimer hydrate of 5-FU (**1**) via head-to-tail coupling between the N1- centered radical and C5’-centered radical of 5-FU (Figure 2) [30,31]. An interesting finding is that the N1-C5’-dimer hydrate **1** undergoes radiation-induced reduction under anaerobic conditions and releases the parent 5-FU with a 16% yield (Figure 3). Radiolysis of the dimer hydrate **1** under N_2_O-saturated conditions, where the primary species of hydrated electrons are scavenged by N_2_O, induced no such release of 5-FU, suggesting the reductive splitting of the dimer **1** is operative under anaerobic irradiation. To evaluate the relationship between the molecular structure and the reactivity of the one-electron reductive release of 5-FU in anoxic aqueous solution, a series of 5-fluoro-1-(2’-oxocycloalkyl)uracils (**2–10**) were synthesized (Figure 4) [39].

**Figure 5:**
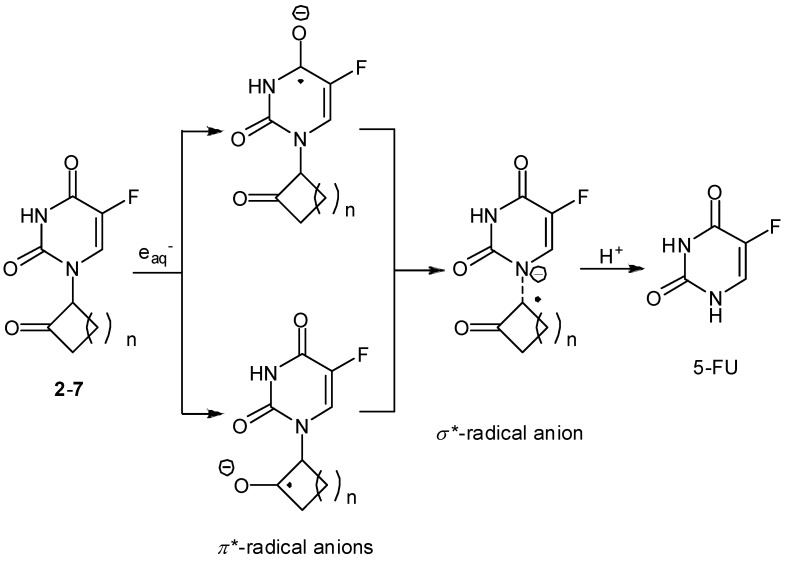
Proposed mechanism for reductive release of 5-FU from **2**-**7**.

All the 5-FU derivatives **2-10** bearing the 2’-oxo group underwent one-electron reduction by the addition of e_aq_^–^ and thereby released 5-FU in sufficient yields of 47-96% upon radiolysis of the anoxic aqueous solution. Release of fluoride ion from the 5-FU derivatives was not observed during the radiolysis suggesting that e_aq_^–^ is captured primarily by the 2’-oxo group. In fact, the control compounds without the 2’-oxo substituent (**11**, **12**) had no reactivity toward such a reductive C1’-N1 bond splitting. Comparing the reactivities of the stereoisomeric derivatives **9** and **10** showed that the efficiency of 5-FU release was strongly dependent on their structural flexibility. X-ray crystallographic studies of representative compounds revealed that the C1’-N1 bond possesses normal geometry and bond length in the ground state. Molecular orbital (MO) calculations by the AM1 method for optimized structures demonstrated that the lowest unoccupied molecular orbital (LUMO) is localized primarily at the π* orbital of the C5–C6 double bond of the 5-FU moiety and that the LUMO + 1 is delocalized between the π* orbital of the 2’-oxo substituent and the σ* orbital of the adjacent C1’–N1 bond (Figure 5). It is presumable that the one-electron reductive release of 5-FU in anoxic aqueous solution occurs from the LUMO + 1 of the radical anion intermediates possessing a partial mixing of the antibonding C(2’)=O π* and C1’–N1 σ* MOs, and that dynamic conformational changes may achieve a higher degree of (π* + σ*) MO mixing.

Based on these observations, our group has developed the simplest prototypes of the 5-FU prodrugs **13**, and we have investigated the radiation chemical reactivity, biological effects, *in vivo* efficacy, pharmacokinetics, and toxicity [40,41]. The prodrug **13** dissolved in phosphate buffer released 5-FU with a G-value (mol number of molecules that are decomposed or produced by 1 J of absorbed radiation energy) of 1.9 × 10^–7^ mol/ J (26%, based on total amount of primary species generated from water) following hypoxic irradiation.

**Figure 6:**
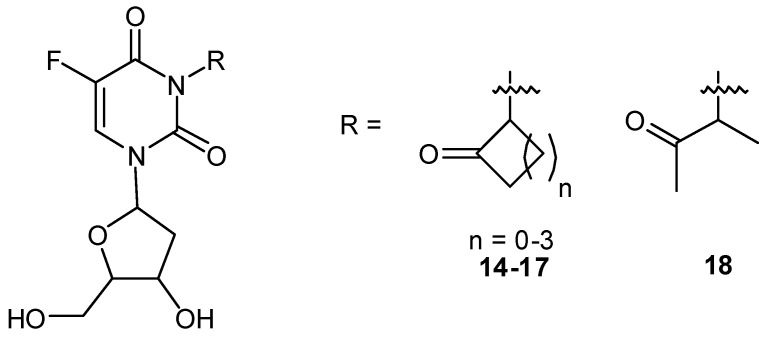
Structures of 5-fluoro-3-(2’-oxoalkyl)-2’-deoxyuridines.

The G-value for 5-FU release was 1.0 × 10^−8^ mol/J following aerobic irradiation. Adding hypoxically irradiated (7.5–30 Gy) **13** to murine SCCVII cells for 1–24 h showed a significant cytotoxic effect. In contrast, cytotoxicity was minimal in cells treated with aerobically irradiated or unirradiated **13**. This compound had no radiosensitizing effect against SCCVII cells under either aerobic or hypoxic conditions when the drug was removed immediately after irradiation. Following administration of **13** and irradiation at 30 Gy, the average 5-FU levels in the tumor and serum were 179ng/g and 83ng/mL, respectively. However, a TCD-50 tumor growth delay assay of **13** demonstrated an enhancement ratio of only 1.2. We recently synthesized a similar family of radiation-activated prodrug of 5-FdUrd [42], which is generally more toxic than 5-FU [43]. 5-FdUrd derivatives **14-18** possessing a 2-oxoalkyl group at the N3 position were synthesized, and their radiolytic one-electron reduction product was characterized (Figure 6).

**Figure 7:**
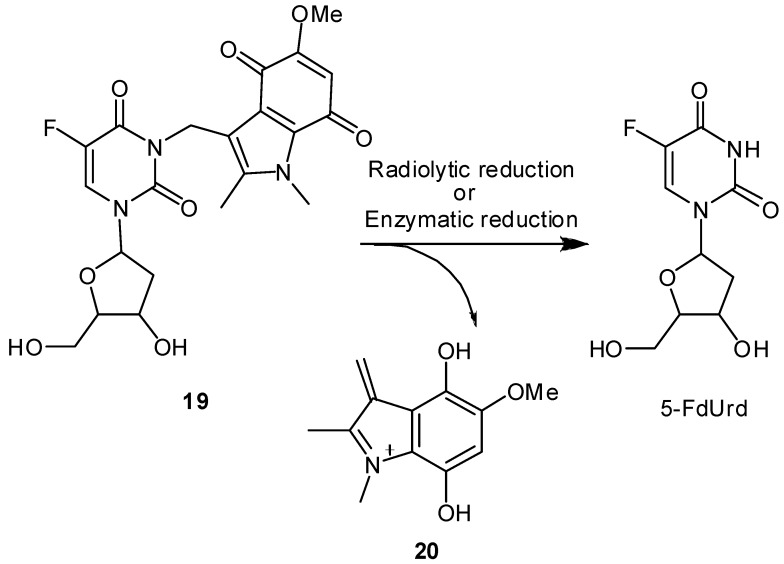
Reductive release of 5-FdUrd from 5-FdUrd-indolequinone prodrug **19.**

The prodrugs released 5-FdUrd efficiently upon hypoxic irradiation by reacting with e_aq_^–^ as in the case of 5-FU. A biological assay using P388 T cells and EMT6/KU cells revealed that hypoxic X-irradiation enhanced cytotoxicity of the prodrugs dramatically. However, we observed no marked effects *in vivo* even when 100 or 300 mg/kg of **16** was combined with 20 Gy of irradiation [44]. Although the *in vivo* effect of both prodrugs of 5-FU and 5-FdUrd was not enough for clinical efficacy, this prototype agent suggests that radiation-activated prodrugs exhibit a certain level of cytotoxicity toward hypoxic tumor cells. As an extension of our work on the development of radiation-activated antitumor prodrugs, we designed another 5-FdUrd prodrug possessing indolequinone structure **19** (Figure 7) [45]. Indolequinone has the following unique reaction characteristics [46,47,48,49]. First, both enzymatic reduction and radiolytic reduction can activate this substituent. Second, the reductive activation of indolequinone derivatives to release drugs is accompanied by the concomitant formation of electrophilic iminium cations like **20**, which may invoke DNA alkylation or other cellular damage [50]. In view of these reaction characteristics, a 5-FdUrd prodrug possessing an indolequinone structural unit may result in synergic cytotoxicity that originates from both the parent drug and electrophilic iminium species released upon radiolytic reduction and bioreduction in hypoxic cells. Hypoxic irradiation of **19** significantly increased the cytotoxicity against hypoxic tumor cells and caused higher cytotoxicity toward EMT6/KU cells than did the hypoxic irradiation with the original 5- FdUrd alone. Our group is currently investigating the *in vivo* effect of **19**.

## Photoactivatable 5-FU Prodrugs

Much research effort has focused on the development of antitumor prodrugs with functions that are activated upon controlled photoirradiation [51]. One general strategy to achieve photoinduced activation of the prodrugs is the initial deactivation of a parent drug by introduction of a photolabile protecting group at a critical position to reduce the cytotoxicity of the chemically modified parent drug under unirradiated conditions. Under photoirradiation with appropriate wavelength, the photolabile protecting group is removed from the prodrug constitution to release a parent drug, which regenerates the original cytotoxicity. Various photoactivated prodrugs with effective photolabile protecting groups (e.g., *o*-nitrobenzyl chromophores, 3,5-dimethoxybenzoin derivatives, and coumarin derivatives) have been developed that can be activated efficiently by irradiation with UV-A (320-400 nm) or visible light to release the parent drugs, including phosphoramidite mustard [52], L-leucyl-L-leucine methyl ester [53,54], aspirin [55,56], cyclic enediyne [57], and paclitaxel [58,59]. Photoactivated prodrugs can be applied in surface cancer treatment and can even be adapted for deep-seated cancer therapy employing endoscopes or optical fibers, as used in photodynamic therapy.

**Figure 8:**
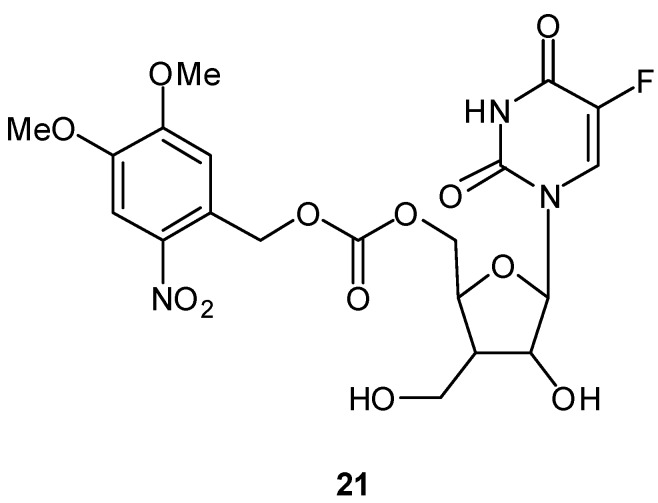
Structure of 5-FdUrd prodrug with the photolabile 4,5-dimethoxy-2-nitrobenzyl group.

Among the various photosensitive protective groups, *o*-nitrobenzyl chromophore [60] is used most commonly for providing photoactivated prodrugs. *o*-Nitrobenzyl is a general photosensitive protective group for various chemical functionalities, such as alcohols, aldehydes, carboxylic acids, amines, and phosphates. Pei and Gong reported recently on a 5-FdUrd prodrug possessing the photolabile 4,5- dimethoxy-2-nitrobenzyl group **21** (Figure 8) [61]. They demonstrated the efficient photoinduced release of 5-FdUrd and 4,5-dimethoxy-2-nitrosobenzaldehyde via a spontaneous decarboxylation reaction from the prodrug **21** by first-order kinetics with a t_1/2_ = 6 min. They also showed inhibition of cell growth by photoirradiation. Without photoirradiation, prodrug **21** only slightly inhibited growth, whereas cell growth was inhibited completely when the cell culture containing prodrug **21 ** was irradiated with a 350 nm lamp.

**Figure 9:**
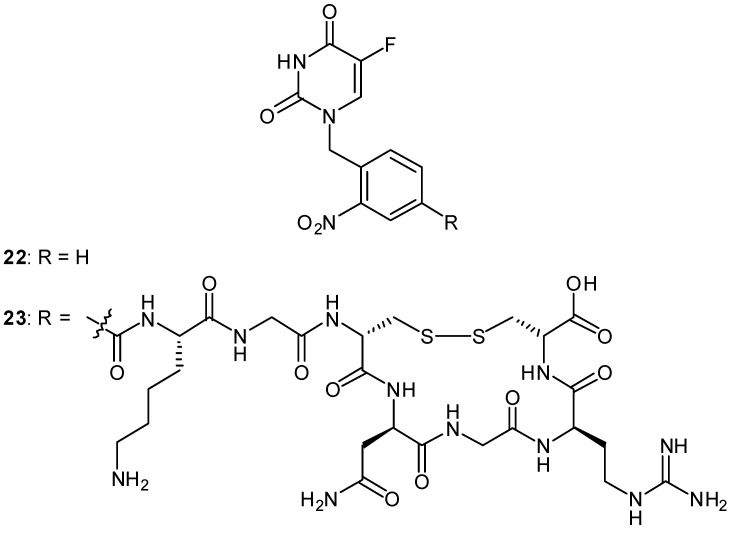
Structures of photoactivated prodrugs containing 5-FU.

We recently designed a photoactivated prodrug of 5-FU conjugated with a tumor-homing cyclic peptide to generate the first prototype compound of a tumor-targeting photoactivated antitumor prodrug **23** (Figure 9) [62]. The tumor vasculature is morphologically abnormal and carries various types of tumor molecular markers that can be used to discriminate tumor vessels from the normal vasculature. We employed chemical conjugation of a 5-FU prodrug possessing an *o*-nitrobenzyl group with cyclic peptide Cys-Asn-Gly-Arg-Cys (CNGRC), which should target one of the tumor molecular markers, a specific APN/CD13 aminopeptidase N. Photoirradiation of the photoactivated prodrug **23** readily removed the photolabile *o*-nitrobenzyl chromophore to give quantitative amounts of 5-FU, whereas the prodrug was quite stable under dark conditions. We also clarified the photoactivation mechanism of the simple model compound **22** using nanosecond laser flash photolysis studies. Although the *in vitro* and *in vivo* evaluation is in progress, the prodrug **23** is expected to show selective cytotoxicity toward tumor tissue upon photoirradiation.

**Figure 10:**
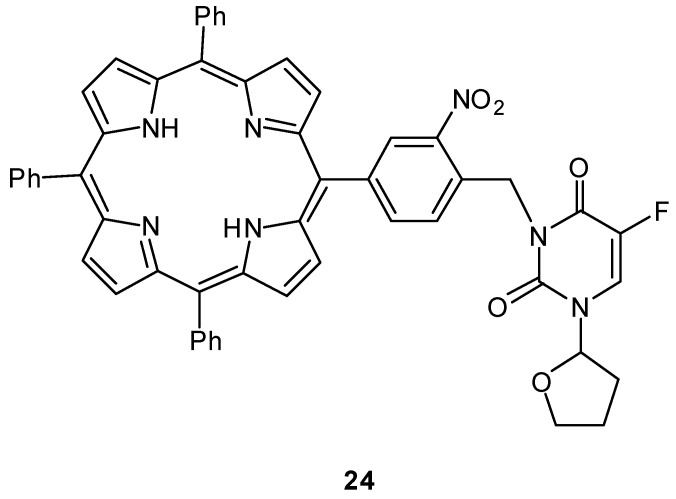
Structures of a photoactivated prodrug containing 5-FU analog **24**.

Lin and coworkers reported another example of a photoactivated prodrug containing 5-fluoro-1- (tetrahydro-2-furanyl)-2,4(1H,3H)-pyrimidinedione (tegafur/Ftorafur) **24** (Figure 10) [63]. Tegafur, an orally active prodrug of 5-FU, is used widely in the treatment of gastrointestinal malignancies and has modest efficacy [64]. Upon photoirradiation at 350 nm, the tegafur prodrug incorporating a triphenylporphyrin and photolabile o-nitrobenzyl group is converted into the anticancer drug tegafur with the photochemical quantum yield Ф = 0.032. An MTT [3-(4,5-dimethylthiazole-2-yl)-2,5- diphenyl-tetrazolium bromide] assay demonstrated that the porphyrin–nitrobenzene–tegafur conjugate shows photoinduced cytotoxicity for MCF-7 mammary cancer cells because of the efficient release of tegafur upon photoirradiation at 350 nm, whereas the prodrug **24** is significantly less toxic than its parent anticancer drug tegafur in the absence of UV irradiation.

**Figure 11:**
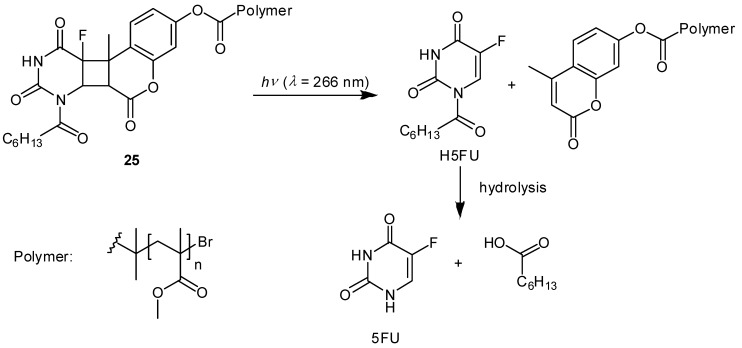
Photo-induced drug release from coumarin-functionalized polymer-H5FU conjugate **25** and subsequent hydrolysis of the prodrug H5FU to 5-FU.

Other interesting examples have been reported. Agarwal and coworkers demonstrated a photoinduced 5-FU release system from methylcoumarin end-functionalized acrylic polymer conjugated with the prodrug 1-heptanoyl-5-fluorouracil (H5FU), which was developed for intraocular lenses equipped with a multidose drug depot used in cataract surgery (Figure 11) [65]. The coumarin-functionalized polymer-H5FU conjugate **25** was synthesized photochemically via a [2+2] cycloaddition reaction between the C3-C4 double bond of methylcoumarin and the C5-C6 double bond of H5FU upon photoirradiation at 350 nm. In contrast to the [2+2] cycloaddition reaction, the [2+2] cycloreversion reaction on the cyclobutane ring proceeds under photoirradiation using a shorter wavelength. Consequently, photoirradiation of 25 at 266 nm induces the cleavage of the cyclobutane linker between the methylcoumarin moiety and the prodrug H5FU, leading to the efficient release of H5FU. Subsequent hydrolysis of H5FU in aqueous solution produces 4.76 μg of 5-FU from 1 mg of the polymer–drug conjugate **25** after photoirradiation for 150 min, indicating that LD50 of 5-FU for rabbit lens epithelial cells can be realized even with small amounts of polymer-drug conjugate **25**, although the photoinduced drug release system has not been evaluated *in vitro* and *in vivo*. Further studies to avoid the risk of DNA being damaged by UV-C radiation would be necessary for its clinical use.

## Conclusions

In the past decade, varieties of photoremovable protecting groups have been developed to control the activity of small molecules, including bioimaging molecules and several kinds of pharmaceutical drugs, by irradiating them with light of specific wavelength and intensity. On the other hand, the ionizing radiation-activated system is not ideal for diagnostic use in the medical field but should be applicable to activating antitumor drugs for sensitizing the drugs to increase the cytotoxic effect of ionizing radiation. In this review, we have summarized the properties of 5-FU prodrugs, which are radiation-chemically or photochemically activatable under the microenvironment of tumor cells. Irradiated prodrugs have high cytotoxicity that is dependent on both the drug contact time and radiation dose, whereas unirradiated prodrugs show minimal cytotoxicity against tumor cells. Disappointingly, the effects of these prototypes, including N1- or N3-(2’-oxoalkyl)-5-FU derivatives, are not strong enough for clinical efficacy, partly due to low efficiency (26% or less) of 5-FU-release from the prodrugs. Although there is limit in clinically permitted does of radiation, we expect that further research on the synthesis and evaluation of more potent anticancer drugs bearing both 5-FU and alkylating agents, such as indolequinone derivatives, will lead to the development of more promising prodrugs that are activatable by external signals.
